# Prevalence of parent-reported food allergy among children in China: A population-based cross-sectional survey

**DOI:** 10.3389/fimmu.2022.982660

**Published:** 2022-12-12

**Authors:** Hua Feng, Nan Luo, Yuanan Lu, Justin Lu, Jiangdong Zhou, Xiujuan Xiong, Zhuo Chen, Yan Chen, Yongning Wu

**Affiliations:** ^1^ State Key Laboratory of Food Science and Technology, Nanchang University, Nanchang, Jiangxi, China; ^2^ School of Public Health, Nanchang University, Nanchang, Jiangxi, China; ^3^ Environmental Health Laboratory, Department of Public Health Sciences, University of Hawaii, Hawaii, HI, United States; ^4^ Fengxin Center for Disease Control and Prevention, Jiangxi, Yichun, China; ^5^ Department of Pathology, Basic Medical College of Nanchang University, Nanchang, Jiangxi, China; ^6^ Sino German Joint Research Institute, Nanchang University, Nanchang, China; ^7^ Research Unit of Food Safety, Chinese Academy of Medical Sciences, National Health Commission (NHC), Key Lab of Food Safety Risk Assessment, China National Center for Food Safety Risk Assessment (CFSA), Beijing, China

**Keywords:** food allergy, prevalence, adverse reactions, questionnaire survey, cross-sectional study

## Abstract

**Objective:**

The prevalence of food allergy (FA) has been increasing in recent years and has become an important public health, food safety, and clinical nutrition problem. However, population-based studies on the prevalence of FA are very limited in China. This study aims to determine the prevalence and pattern of parent-reported FA among school children in Jiangxi Province, China.

**Methods:**

A multicenter cross-sectional study on FA was conducted on primary school children aged 6-11 years old using random cluster sampling with a questionnaire survey. Parent-reported FA was used and defined as individual-reported FA by parent or guardian through a questionnaire in this study.

**Result:**

Among the total of 8,856 (96.36%) complete questionnaires received, 727 (8.2%) children had adverse reactions to food (ARF). The prevalence rates of parent-reported FA and doctor-diagnosed FA were 6.2% and 3.3%, respectively. Animal-derived foods were the main causative source of FA, and the three leading allergenic foods were shrimp, mango, and mollusks. Skin reactions were the most common clinical manifestations of FA, accounting for 63.7%, and 45.32% of the subjects with parent-reported FA experienced severe allergic reactions. There was a significant difference in parent-reported FA between different survey centers, and FA risk increased significantly in children with other allergic diseases (*P*<0.001) and small family size (*P*=0.026). The FA prevalence was significantly higher among children aged 8-11 years than those aged 6-7 years (*P*=0.020).

**Conclusions:**

A high prevalence of parent-reported FA was observed among children in general primary schools in Jiangxi Province, China. Shrimp, mango, and mollusks were the most common causative foods. The main common symptoms of FA were adverse reactions relating to the skin system. The rate of severe allergic reactions was also high in Jiangxi Children with reported FA. Local standards and policies for the prevention and management of FA need to be adjusted on a timely basis according to actual local conditions.

## Introduction

Food is an indispensable part of life; however, improper diets can cause nutrition, health, and food safety problems. Some studies have shown that up to 41.6% of individuals reported adverse reactions to food (ARF) (1). One of the ARFs is food allergy (FA). In childhood, food allergic reactions are more common compared to nonallergic food hypersensitivity reactions (2).

Food allergy is a complex immune-mediated disorder that affects all ages, especially children. Based on numerous epidemiological studies, FA has become a common chronic disease in children (3) and affects approximately 8% of children (4–8), and even up to 10% in developed countries (9). The prevalence of FA is increasing globally (10–14) and the types of foods causing allergies and serious allergic reactions are growing (2, 15). Therefore, FA is described as the “second wave” of allergic disease after asthma and allergic rhinitis (16).

Food allergy is known to be associated with a negative impact on child growth and psychosocial condition, serious impairment of their quality of life, and is also the main cause of anaphylaxis among patients presenting to hospital emergency departments (10, 17–19). FA not only impacts health, psychological well-being, nutritional status, and the economy (20), but is also an important issue relating to clinical nutrition, food safety, and public health.

The prevalence and trend of FA in the community largely depend on public awareness, diagnostic methods, and dietary habits of the population (21), and vary among different geographic regions. This issue has been well documented in high-income countries (HIC), but information available about it is very limited in many low- and middle-income countries (LMIC) (22). The epidemiology of potentially life-threatening FA in Asia is unclear (23). Currently, few epidemiological studies on FA have been conducted and there is a lack of information from large-scale population-based studies on FA in China. In addition, a small-scale research study reported that the prevalence of confirmed FA in children was approximately 6% in China (24), indicating a consistent upward trend with global prevalence (16). A recent meta-analysis of the Chinese population has also concluded that FA prevalence from 2009–2018 was 8% (95% CI: 6%–11%), higher than that from 1999-2008 (5%; 95% CI: 3 %–7%) (25).

Studies on FA in the general population with a large sample size have not been done in Jiangxi Province. Jiangxi, one of the inland provinces in China, is located in the central southeast of China and covers an area of 166,900 square kilometers. Jiangxi is economically ranked in the middle of the country and accounts for a large share of agriculture, with grain, oilseeds, and vegetables. Jiangxi is a provincial administrative division with 11 prefecture districts and 100 counties, a population of 45.18 million, and 55 ethnic groups, of which the Han account for more than 99%. There are about 7,200 elementary schools with 242,200 teachers and 4,063,100 students.

This survey-based study was designed to estimate the prevalence and pattern of parent-reported FA, identify main foods allergens and understand the most common symptoms of FA. This study also included the prevalence of doctor-diagnosed FA, and the other allergic diseases among children in Jiangxi, China. Findings from this study will form the baseline not only for improving current awareness of FA among children and their parents but also for facilitating the development of an effective strategy to prevent and control FA among school children.

## Subjects and methods

### Study population

**Figure 1:**
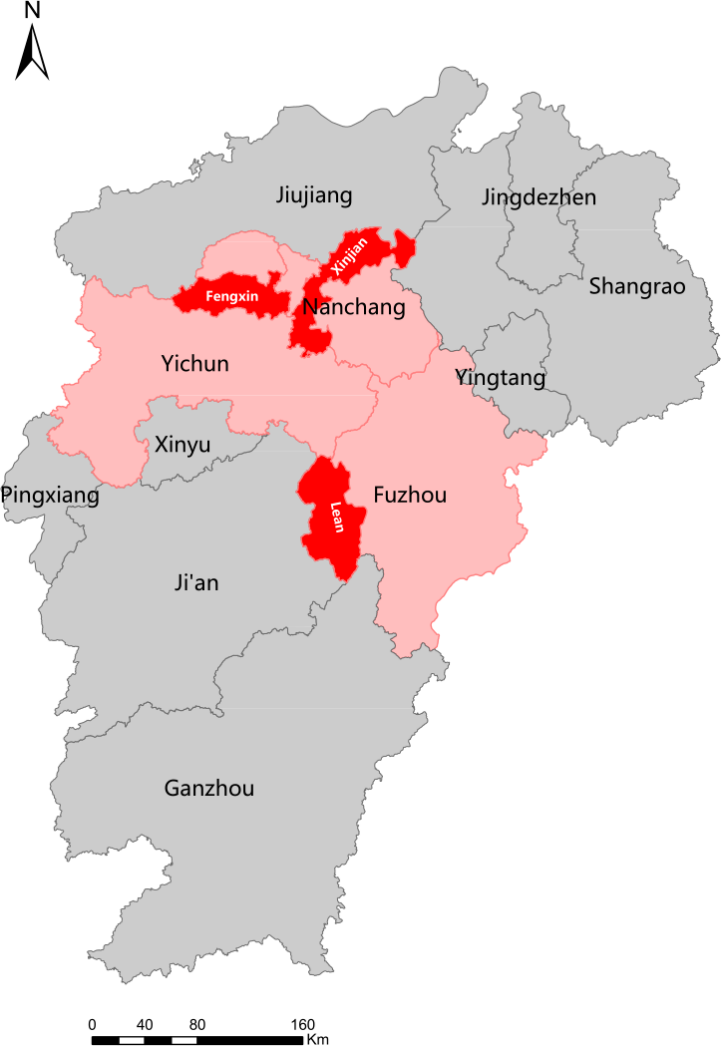
Distribution of survey centers.

A population-based cross-sectional epidemiological survey on FA was conducted among more than 9000 children using stratified cluster random sampling in Jiangxi Province from January 2020 to May 2021. Three prefectures were randomly selected in Jiangxi and then one district or county was randomly chosen from each selected prefecture as the survey center (**Figure 1**). The subjects were children aged 6-11 years currently enrolled in primary schools. First, the lists of primary schools were obtained from local education departments, then elementary schools from rural and urban regions were randomly selected. Approximately 3,000 schoolchildren were recruited from primary schools in each survey center using class-based random cluster sampling. A total of 9,000 representative children from 16 primary schools in three survey centers were enrolled in this study. Approximately half of them came from rural regions and the others came from urban regions.

### Questionnaire

The questionnaire designed for this study was mainly based on the EuroPrevall FA questionnaire (26) with some modifications according to a validation test to accommodate the indigenous need of the local people. Several questions about demography, ARF, FA, doctor-diagnosed FA, and other allergic diseases were the focus of this questionnaire.

### Survey method

All the children aged 6-11 years old in grades 1 to 5 from each selected school were invited to participate in this study. The questionnaires were distributed, in the school classes, to their parents or legal guardians for completion. The class teachers and investigators collected and checked the filled questionnaires. Questionnaires with any problems such as incomplete or unclear answers were then returned to respondents for clarification and completion.

### Study management

The expert panel of this study formulated and approved standard survey procedures, and all staff were well-trained in the standard procedures and methods and conducted the survey accordingly. The survey data was cross-checked and verified independently by two individuals and was then used to form a database *via* a web APP (Information Management System of Chinese FA Epidemiological Investigation), which was specifically designed for data capturing in this project.

### Definition

Several definitions used in this study were based on the descriptions published previously (27). ARF was defined as the report of an illness or disorder after consuming food: parent-reported FA was defined as FA reported by participants’ parents or guardians, doctor-diagnosed FA referred FA diagnosed by doctors, and multiple FA meant allergies to 3 or over 3 food allergens.

### Statistical analysis

The descriptive statistics for categorical variables were presented as frequencies and percentages, while continuous variables were summarized as means ± standard deviation (SD). Pearson’s chi‐squared or Fisher’s exact tests were used for categorical variables. Comparisons of continuous variables were performed with a t-test or one-way ANOVA. The statistical analysis was performed with SPSS version 25.0, and two-tailed *P* values of <0.05 were considered as statistical significance.

### Ethical approval

This study was approved by the Ethical Committees of the China Center for Food Safety Risk Assessment (Number 2019027) and written informed consent was obtained from each child’s legal guardian.

## Results

### Participants

A total of 9,191 questionnaires were randomly distributed to schoolchildren in 16 primary schools from urban and rural regions in Jiangxi, China. Among the 8,856 (96.36%) valid questionnaires collected, 4,658 were from boys, 4,198 were from girls, 4,141 were from urban children, and 4,715 were from rural children. The mean age and height of children were 8.67 ± 1.26 years old and 1.31 ± 0.10 meters, respectively. **Table 1** shows the demographic characteristics of the participants.

**Table 1:** Demographic characteristics and prevalence of reported ARF and FA.

Variables		Total( n, %) /Mean ± SD	AFR ( n, %) /Mean ± SD	FA ( n, %) /Mean ± SD	Non-FA( n, %) /Mean ± SD	*X* ^2^/t value	*P* value
**Total**		**8856 (100)**	727 (8.2)	**545 (6.2)**	**7834 (88.5)**	–	–
**Gender**	Male	**4658 (52.6)**	392 (8.4)	297 (6.4)	4117 (88.4)	0.771	0.380
	Female	**4198 (47.4)**	335 (8.0)	248 (5.9)	3717 (88.5)		
**Age (year)**		**8.7 ± 1.26**	8.8 ± 1.27	8.8 ± 1.28	8.7 ± 1.25	1.787	0.074
**Age group**	6-7	**2861 (32.3)**	207 (7.2)	159 (5.6)	2513 (87.8)	7.869	**0.020**
	8-11	**5995 (67.7)**	652 (10.9)	386 (6.4)	5321 (88.8)		
**BMI**		**16.6 ± 3.5**	16.7 ± 3.56	16.7 ± 3.33	16.6 ± 3.57	0.924	0.355
**Ethnic**	Han	**8825 (99.6)**	724 (8.2)	542 (6.1)	7834 (88.8)	0.344	0.558
	Others	**31 (0.4)**	3 (9.7)	3 (9.7)	24 (77.4)		
**Family size**		**5.1 ± 1.53**	5.1 ± 1.43	5.0 ± 1.44	5.2 ± 1.54	-2.23	**0.026**
**Residence**	Urban	**4141 (46.8)**	339 (8.2)	279 (6.7)	3916 (94.6)	1.879	0.170
	Rural	**4715 (53.2)**	388 (8.2)	266 (5.6)	4215 (89.4)		
**Survey center**	Fengxin	**2760 (31.2)**	267 (9.7)	210 (7.6)	2412 (87.4)	19.534	**0.000**
	Lean	**3099 (35.0)**	252 (8.1)	189 (6.1)	2688 (86.7)		
	Xinjian	**2997 (33.8)**	208 (6.9)	146 (4.9)	2734 (91.2)		
**Other allergies (except FA)**		**664 (7.5)**	295 (40.6)	242 (44.4)	363 (4.6)	1203.051	**0.000**
	Allergic dermatitis	**330 (3.7)**	98 (13.5)	148 (27.2)	155 (2.0)	926.735	**0.000**
	Allergic rhinitis	**278 (3.1)**	28 (3.9)	75 (13.8)	179 (2.3)	228.317	**0.000**
	Allergic asthma	**56 (0.6)**	33 (4.5)	22 (4.0)	28 (0.4)	116.288	**0.000**

FA, food allergy; ARF, adverse reactions to food. The bold values mean the data in total or showing statistic difference (P < 0.01).

### Prevalence of parent-reported ARF and FA

**Figure 2:**
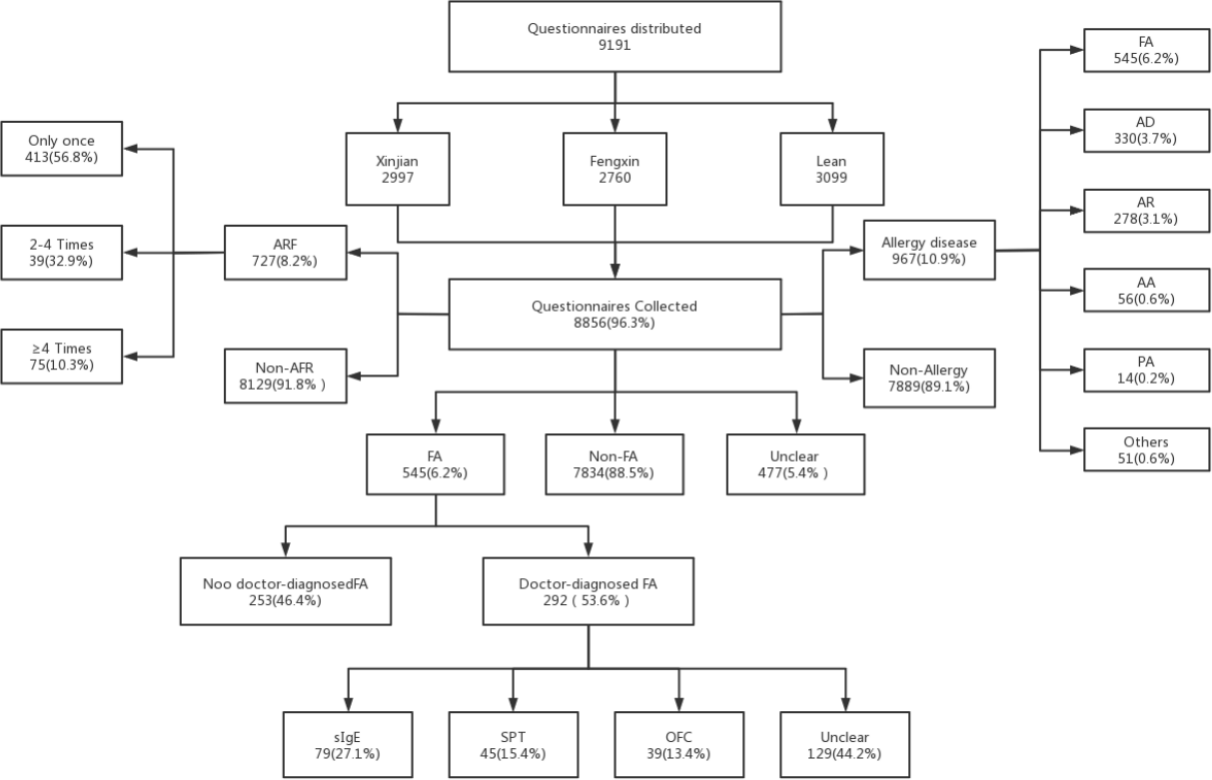
Overview of participation, distribution of ARF, and allergic diseases.

Our study showed no age difference between the ARF group and the non-ARF group. Among the participants, 727 reported AFR with a mean age of 8.79 ± 1.27, 56.8% experienced ARF only once, and 10.3% experienced it four or more times. Among the non-ARF children (8,129), the mean age was 8.66 ± 1.25. A total of 545 children (6.2%) reported an FA, 88.5% (7,834) reported no FA, and 5.4% (477) did not know if they had an FA. **Table 1** summarizes the prevalence of parent-reported ARF and FA. There was no significant difference in FA prevalence between gender, ethnicity, residence, or BMI. However, FA was associated significantly with age group, family size, living region, and other allergic diseases. FA prevalence among 8-11-year-old children was higher than that in 6-7-year-old children (*P*=0.020), and FA was more common in children with other allergic diseases (*P*<0.001) or small family size (*P*=0.026). **Figure 2** indicates the overview of participants, distribution of ARF, and allergic diseases. There were 292 doctor-diagnosed FA cases (3.30%) reported, accounting for 53.58% of the parent-reported FAs, of which 15.41% suffered multiple FAs.

### Food allergens and symptoms of FA

This survey revealed over 100 food allergens and animal-derived foods were dominant. As shown in **Table 2**, the most common causative foods were shrimp, followed by mango, mollusks, egg, fish, milk, beef, and lamb. Peanuts were not included in the top eight food allergens. The top three leading food allergens accounted for 39.45%, 30.28%, and 22.57%, respectively. Mango, beef, and lamb were determined to be among the top eight food allergens in this study, but they are not included in the recommended list of food allergens in the Chinese GB 7718-2011 National Food Safety Standard. In total, 77 children (0.87%) were reported with multiple FAs, accounting for 14.13% of parent-reported FAs. **Table 3** lists the major causative foods of self-reported doctor-diagnosed FA, and it also presents mango, beef, and lamb as the common causative foods.

**Table 2:** Summary of the common food allergens of parent-reported food allergy.

Food Allergens	n	Proportion %	Prevalence %	Included in GB 7718
**Shrimp**	215	39.45	2.43	Yes
**Mango**	165	30.28	1.86	**No**
**Mollusks**	123	22.57	1.39	Yes
**Egg**	110	20.18	1.24	Yes
**Fish**	65	11.93	0.73	Yes
**Milk**	53	9.72	0.60	Yes
**Beef**	41	7.52	0.46	**No**
**Lamb**	34	6.24	0.38	**No**
**Peanut**	30	5.50	0.34	Yes
**Soybean**	19	3.49	0.21	Yes
**Tree Nuts**	15	2.75	0.17	Yes
**Sesame**	15	2.75	0.17	**No**
**Crab**	12	2.20	0.14	Yes
**Wheat**	9	1.65	0.10	Yes
**Peach**	5	0.92	0.06	**No**
**Pineapple**	5	0.92	0.06	**No**

The bold values mean that food allergens are not included in Chinese GB 7718.

**Table 3:** Causative foods of the parent-reported doctor-diagnosed food allergy.

Causative Foods	FA (n)	FA Prevalence (%)	FA Proportion (%)
Total	292	3.30	53.58
**Shrimp**	95	1.07	32.53
**Mango**	60	0.68	20.55
**Egg**	57	0.64	19.52
**Mollusks**	54	0.61	18.49
**Milk**	33	0.37	11.30
**Fish**	31	0.35	10.62
**Beef**	24	0.27	8.22
**Peanut**	20	0.23	6.85
**Lamb**	14	0.16	4.79
Soybean	11	0.12%	3.77
Crab	9	0.10	3.08
Nuts	6	0.07	2.05
Sesame	5	0.06	1.71
Wheat	4	0.05	1.37

**Figure 3:**
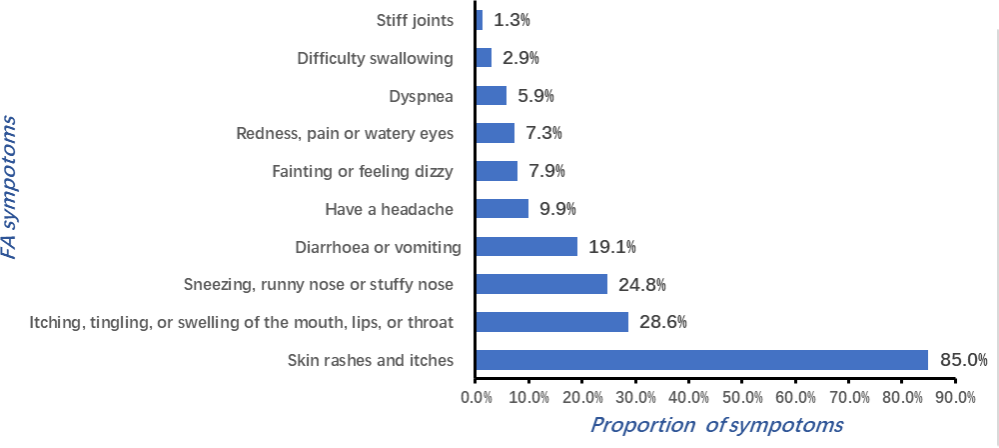
Reaction characteristics and common symptoms of FA.

**Figure 4:**
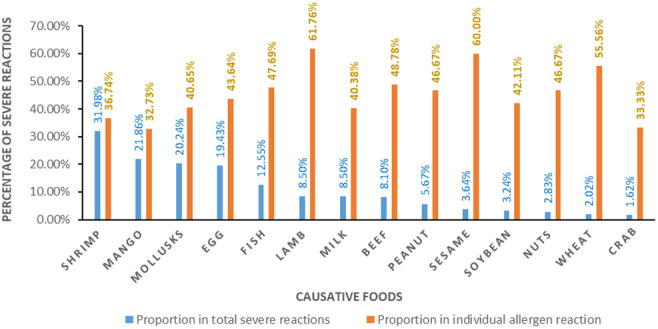
Severe reactions caused by major allergens.

This survey showed that the main clinical manifestations of FA were diverse and heterogeneous, primarily associated with the skin, perioral, respiratory tract, digestive tract, and cardiovascular system. Skin symptoms were particularly prominent, such as rashes and itches, accounting for 85% of the FA reaction. Perioral symptoms accounted for 28.6% of FA cases, while 18% of FA cases presented headaches and dizziness, as shown in **Figure 3**. Among the subjects with parent-reported FA, more than 45% (247) experienced severe allergic reactions, among them 12.41% were allergic to multiple foods. **Table 4** shows the common symptoms caused by major allergens, and the severe reactions caused by major food allergens are presented in **Figure 4**.

**Table 4:** The prevalence of main clinical symptoms associated with major allergenic foods (%).

Symptoms	Egg	Milk	Peanut	Fish	Shellfish	Mollusks	Mango	Beef	Mutton
Oropharyngeal symptoms	13.50	11.40	26.30	17.60	20.10	19.40	35.20	12.50	23.80
Skin symptoms	79.80	68.20	73.70	72.50	70.70	72.80	71.00	75.00	71.40
Gastrointestinal symptoms	14.60	29.50	36.80	11.80	12.00	20.40	9.00	21.90	23.80
Nasal symptoms	14.60	22.70	10.50	15.70	16.80	19.40	12.40	34.40	47.60
Eye symptoms	5.60	2.30	15.80	7.80	7.10	7.80	7.60	15.60	28.60
Difficulty swallowing	2.20	4.50	5.30	3.90	2.20	1.00	1.40	6.30	4.80
Dyspnea	3.40	6.80	10.50	7.80	4.90	5.80	2.10	9.40	9.50
Stiff joints	2.30	1.10	5.30	2.00	1.60	1.00	0.70	3.10	4.80
Fainting and dizziness	5.60	4.50	5.30	7.80	6.00	9.70	4.80	18.80	19.00
Headache	4.50	6.80	10.50	13.70	3.30	4.90	4.10	12.50	23.80

## Discussion

Food allergy is affecting the growth and development of children. A recent study from Australia has suggested that the rate of admissions for food anaphylaxis is increasing, and more dominant among elementary and secondary school children than among preschoolers (28). The prevention and treatment of FA are becoming a challenge for doctors, scientists, politicians, and the public (2). Allergen avoidance and drugs in case of an allergic reaction currently remain the standard of care for FA (14). Therefore, a clear understanding of the characteristics and patterns of FA prevalence will help to improve public health services and the prevention and management of FAs.

### Prevalence of parent-reported FA

This study showed that 8.2% of parent-reported AFR prevalence among primary school children was higher for Asian children, which previously ranged between 1.2% and 6.5% (23), but was lower than the 10.5% prevalence of hypersensitivity to food among children in Italy, 46.9% among primary school children in Vilnius, Lithuania (2), and 12.6% in Japan (6), but was consistent with the 8% in the United Arab Emirates (2) and 8.1% in Hong Kong (23). The prevalence of parent-reported FA was estimated to be 6% among primary school-age children in Turkey and 7.6% in the USA (29), which are in accordance with our findings. The dietary culture, living environment, and genetic susceptibility are diverse in different countries or regions which might contribute to different distributions and features of FAs in different regions and countries.

### Food allergens

Previous studies reported that the most common food allergens responsible for clinical reactions included milk, egg, peanut, tree nuts, mollusks, fish, wheat, and soy, which accounted for 90% of serious allergic reactions (6, 8). The list and order of the common food allergens vary in different countries or regions. For example, fish is a more common food allergen in Scandinavia; while soy is more common in Japan; peanuts in the United States and the United Kingdom; tropical fruits in Southeastern Asia; and seafood in Mediterranean countries. The different observations are most likely due to the differences in cultural traditions, dietary habits, culinary practices, and genetic factors (2).

Our study revealed some common food allergens for both parent-reported FA and doctor-diagnosed FA. Among the 100 FA causative foods analyzed, the top eight offending foods are different from the reports of FA studies in Hong Kong (23) and other regions (2, 29). The prevalence of peanut allergy is as high as 1%-3% among children in the USA, UK, and Australia but is less than 1% in most Asian countries (20, 30). Our study demonstrated that the prevalence of peanut allergy was 0.34%. Consuming peanuts in the form of boiled peanuts and peanut oil are very common in Southern China. The EuroPrevall-INCO Survey revealed that the prevalence of peanut allergy was 10 times higher in British Jewish children than in Israeli Jewish children, suggesting that high peanut consumption in early life could play an important role in explaining the difference (15).

The GB 7718-2011 National Food Safety Standard-General Standard for the Labeling of Prepackaged Foods in China lists eight categories of allergenic substances, referring to the Codex Alimentarius Standard, of recommended foods to encourage enterprises to voluntarily label to inform consumers. Three of the top eight allergenic foods (mango, beef, and lamb) in our study are not in the eight recommended food allergens list of GB 7718-2011. They were also the major food allergens that can cause severe allergic reactions. Since the occurrence and development of FA may change with economic and social development, relevant local standards should be adjusted on a timely basis according to the local situation in order to prevent and manage FA effectively.

### Common symptoms of FA

Food allergy is a potentially fatal immune disease (31). Living with FA in childhood is well-recognized as impairing health (32, 33).

This study showed that skin symptoms were particularly prominent, such as rashes and itches, and accounted for 85% of the FA reaction. Perioral symptoms, nasal symptoms, headache, and dizziness were also noted frequently. Ebisawa and their team demonstrated that the most frequent clinical manifestations of FA were skin symptoms (92.0%), followed by respiratory symptoms (33.6%), mucosal symptoms (28.0%), gastrointestinal symptoms (18.6%), and shock (10.4%) (34). However, a similar study concluded that diarrhea and vomiting were the most common reactions of FA among schoolchildren (48.0%); a rash, urticarial rash, or itchy skin were also frequently observed disorders (46.4%) (2). Our study substantiated the previous findings and showed a high rate of severe allergic reactions (>45%) among the children with parent-reported or doctor-diagnosed FA.

### Possible risk factors of food allergy

The epidemiological characteristics of FA may be related to the complex interaction of polygenic inheritance and environmental factors. The prevalence of FA ranges from 2% to 10%, based on different variables such as age, geographical location, or ethnicity (2, 29), and declines with the increase in age (22). However, a meta-analysis including 24 studies with 138,740 subjects concluded that the prevalence of FA in children aged 4-17 years was the highest (10%; 95% CI: 7%–14%) in China (25). Our survey study showed that the FA prevalence in schoolchildren was lower compared with that reported in teenagers (12.0%) (21) and college students (15.7%) (35). The FA prevalence among 8-11-year-old children was significantly higher than that among children aged 6-7 years old. Hayashi concluded that the FA prevalence was significantly higher in boys than girls among the schoolchildren (3), but our finding does not support this observation and instead is more consistent with the study among 7–15-year-old children in Japan showing the FA prevalence of 6.3% without difference between boys and girls (3). A previous report noted ethnic differences in the prevalence of FA among African Americans (10); the small sample size of ethnic minorities in this study prevented the assessment of such differences here. Levin et al. reported that exposure to livestock in rural regions could be the strongest protective factor against FA, and the prevalence of allergic diseases was higher in urban regions than in rural regions among South African children (36). This survey showed no difference in FA prevalence between rural children and urban children. A possible explanation may be due to the progress of urbanization in China and the diminishing lifestyle difference between urban and rural regions. Since the living environment and diet habits are quite different among the three survey centers, this may explain the variation in the FA prevalence among them. For example, kiwi is a locally special fruit of Fengxin and known to be an allergen there, however, we found that no child was allergic to it in the other two survey centers. According to the hygiene hypothesis, large family size is a protective factor against FA (17), and our study also showed that the prevalence of FA decreased with an increase in family size.

Food allergy is often the first clinical appearance of allergy and the first link of the so-called “march of atopy” (2). It is also associated with a higher risk of severe asthmatic forms in children (37) and more than a third of food-allergic children have asthma (38). Our analysis has also demonstrated that the prevalence of other allergic diseases was higher in children with FAs than in those without FAs. This finding was in agreement with the result reported by Liu et al. (39) Therefore, FA may be a marker of a generalized phenotype for atopic diseases, which may suggest that atopic diseases coexist and may share the same susceptibility genes (10).

This study has several limitations. It is a cross-sectional study, so it could not confirm a causal relationship and the incidence of FA was not available. Another major limitation is that our study was based on parent-reported FA. Recall bias may exist even though many common offending foods were listed in the questionnaire for parents to choose from and retrospective information about the FAs of their children. Some of the reported cases will not be substantiated when clinical confirmation is sought, because other ARF such as food intolerance may be regarded as FAs, so self-reported FA may overestimate FA prevalence by three or four times (40, 41). On the other hand, parent or self-reported FA is affected by many factors, such as economic status, awareness of FA, medical resources, etc., and people may be unaware of FAs or mistake an FA for another ARF, then result in the underestimation of FA prevalence. One study found that approximately 30.0% of allergic reactions occurred in children who were not previously known to have an FA (4). The Food Allergy Working Group of the Latin American Society for Pediatric Gastroenterology, Hepatology and Nutrition recently published the results of a survey that showed much diversity in the diagnostic approach, as well as poor adherence to the existing guidelines (42), but the consensus is that self-reported FA can be an appropriate method for assessing FA prevalence in population-based studies if ancillary testing is not possible (10). It is the initial evidence and an important first step for studies on FA prevalence, so self-reported FA is usually used in studies on FA, especially in a large-scale general population. Nevertheless, this is the first large-scale multicenter study on FA among children with a higher participation rate, high effective response rate, and compliance in Jiangxi Province, China. Strict quality control work was performed during the survey. The result will help to improve awareness of FA in the population and provide insights about the epidemiological characteristics of FA in China, which may form the scientific basis essential for the development of effective approaches for the prevention and control of FA in Jiangxi, and even in wider China. More objective diagnoses of FA including SPT, sIgE, and OFC will be performed in a further study to confirm these findings. The protocol is available in China CDC Weekly (27).

## Conclusion

Our study has clearly demonstrated that the prevalence of parent-reported FA is not low among school children in Jiangxi Province, China. More than 45% of the children with parent-reported FA had experienced severe allergic reactions. Three of the eight leading food allergens are not included in the recommended eight food allergens in Chinese GB 7718. These findings suggest that bringing public attention to the importance of carefully reading food allergen labels is necessary to avoid food allergens.Furthermore, health education on FA prevention and treatment should be implemented for school administrators, teachers, medical care personnel, parents, and children in order to increase their awareness and essential knowledge of FA and effectively prevent and control FA in school children. In addition, local standards and policies for preventing and managing FA should be adjusted on a timely basis according to local conditions.

## Data availability statement

The original contributions presented in the study are included in the article/supplementary material. Further inquiries can be directed to the corresponding authors.

## Ethics statement

This study was approved by the Ethical Committees of China Center for Food Safety Risk Assessment and written informed consents were obtained from each child’s legal guardian.

## Author contributions

HF, NL, YW, YC conceived and coordinated the study, designed, performed and analyzed the experiments, wrote the paper. YL, JL, JZ and XX, ZC carried out the data collection, data analysis, and revised the paper. All authors contributed to the article and approved the submitted version.
